# 5-Methoxyflavone-induced AMPKα activation inhibits NF-κB and P38 MAPK signaling to attenuate influenza A virus-mediated inflammation and lung injury in vitro and in vivo

**DOI:** 10.1186/s11658-022-00381-1

**Published:** 2022-09-30

**Authors:** Sushan Yang, Linxin Wang, Xiping Pan, Yueyun Liang, Yuehan Zhang, Jing Li, Beixian Zhou

**Affiliations:** 1 The People’s Hospital of Gaozhou, Gaozhou, 525200 China; 2 Guangzhou Laboratory, Guangzhou, China; 3 State Key Laboratory of Respiratory Disease, National Clinical Research Center of Respiratory Disease, Guangzhou Institute of Respiratory Health, The First Affiliated Hospital of Guangzhou Medical University, Guangzhou Medical University, Guangzhou, China; 4 Institute of Chinese Integrative Medicine, Guangzhou Medical University, Guangzhou, Guangdong China

**Keywords:** 5-Methoxyflavone, Influenza A virus, AMPKα, Anti-inflammatory, Antiviral

## Abstract

Influenza-related acute lung injury (ALI) is a life-threatening condition that results mostly from uncontrolled replication of influenza virus (IV) and severe proinflammatory responses. The methoxy flavonoid compound 5-methoxyflavone (5-MF) is believed to have superior biological activity in the treatment of cancer. However, the effects and underlying mechanism of 5-MF on IV-mediated ALI are still unclear. Here, we showed that 5-MF significantly improved the survival of mice with lethal IV infection and ameliorated IV-mediated lung edema, lung histological changes, and inflammatory cell lung recruitment. We found that 5-MF has antiviral activity against influenza A virus (IAV), which was probably associated with increased expression of radical *S*-adenosyl methionine domain containing 2 (RSAD2) and suppression of endosomal acidification. Moreover, IV-infected A549 cells with 5-MF treatment markedly reduced proinflammatory mediator expression (IL-6, CXCL8, TNF-α, CXCL10, CCL2, CCL3, CCL4, GM-CSF, COX-2, and PGE_2_) and prevented P-IKBα, P-P65, and P-P38 activation. Interestingly, we demonstrated that 5-MF treatment could trigger activation of AMP-activated protein kinase (AMPK)α in IV-infected A549 cells, as evidenced by activation of the AMPKα downstream molecule P53. Importantly, the addition of AMPKα blocker compound C dramatically abolished 5-MF-mediated increased levels of RSAD2, the inhibitory effects on H1N1 virus-elicited endosomal acidification, and the suppression expression of proinflammatory mediators (IL-6, TNF-α, CXCL10, COX-2 and PGE_2_), as well as the inactivation of P-IKBα, P-P65, and P-P38 MAPK signaling pathways. Furthermore, inhibition of AMPKα abrogated the protective effects of 5-MF on H1N1 virus-mediated lung injury and excessive inflammation in vivo. Taken together, these results indicate that 5-MF alleviated IV-mediated ALI and suppressed excessive inflammatory responses through activation of AMPKα signaling.

**Supplementary Information:**

The online version contains supplementary material available at 10.1186/s11658-022-00381-1.

## Introduction

The onset of acute lung injury (ALI) triggered by influenza A virus (IAV) infection is rapidly progressive and life threatening, which has been reported as the leading cause of influenza-related deaths [1, 2]. The pathological features of influenza-related lung injury are dominated by pronounced pulmonary edema, diffuse alveolar damage, excessive inflammation, large amounts of exudate (protein-rich fluid), and respiratory failure [3]. Accumulating evidence demonstrates that both viral virulence factors and host immunity are responsible for the acute exacerbation of influenza diseases [4]. As a negative-sense, single-stranded RNA virus, influenza virus (IV) is more prone to undergo genetic mutation and reassortment. Emerging influenza variants rapidly acquire resistance to virus-targeting agents (e.g., M2 channel inhibitors, neuraminidase inhibitors) [5]. Currently, the clinical therapeutic agents for influenza disease treatment are still limited. It is possible that the influenza variants acquire adaptation to sustain transmission among humans, resulting in new global pandemics. Novel intervention strategies for combating future new influenza pandemics are desperately needed.

The transcription factor nuclear factor κB (NF-κB) is ubiquitously expressed in the cytosol of quiescent cells [6]. Upon infection with IAV, virus-derived 5′-triphosphate viral RNA and its structural proteins (e.g., M1, HA, and NP) are believed to be responsible for triggering NF-κB activation [7–9]. NF-κB activation occurs by release from IκBα and subsequent nuclear translocation of NF-κB to drive a number of genes, including adhesion molecules, such as intercellular adhesion molecule (ICAM)-1, vascular cell adhesion molecule (VCAM)-1, and E-selectin, as well as the proinflammatory mediators, interleukin (IL)-6, IL-8, and tumor necrosis factor α (TNF-α) [10]. Since it is also involved in initiating antiviral factor expression (e.g., IFN-β), NF-κB is considered to play an important role in the defense against viral infection [11]. However, NF-κB has been identified as a critical promoter of influenza-related inflammation and efficient viral replication [12]. It has been found that massive inflammation caused by highly pathogenic avian H5N1 viruses is highly dependent on NF-κB [13]. Experiments in vitro and in vivo have proven that inhibition of NF-κB protected against IV-mediated lethal lung injury through inflammation attenuation [14]. Moreover, various studies have revealed that NF-κB signaling was manipulated by IV to support viral replication [12, 15]. During the early stages of IV infection, NF-κB has been found to be essential for supporting viral RNA synthesis [12]. Moreover, later in the IV packaging stage, NF-κB has a critical role in promoting viral ribonucleoprotein (vRNP) nuclear export [15]. Strikingly, activation of NF-κB signaling was reported to impair type I interferon-induced antiviral immunity via elevation of suppressor of cytokine signaling-3 (SOCS-3) [16]. These findings suggest that blockade of NF-κB may be beneficial in reducing IV replication and influenza-related inflammation. Mitogen-activated protein kinases (MAPKs) have a critical role in transducing extracellular stimuli to cellular responses that govern cell proliferation, death, differentiation, and inflammation [17]. Besides NF-κB, MAPKs are also activated in virus-infected cells, which are found to be utilized by various RNA respiratory viruses for their lifecycle and are associated with severe inflammation [18, 19]. The activation of p38 kinase, a member of the MAPK family, was closely correlated with the increasing production of NOX4-derived reactive oxygen species (ROS) in IV-infected cells [20]. Previous studies found that p38 kinase was overactivated in patients with H5N1 virus infection and was responsible for the virus-associated overwhelming inflammation [21]. In regard to supporting viral replication, activation of p38 kinase was required for virus entry into host cells [22]. The increased p38 kinase activity elicited by IV infection facilitated the promotion of vRNP nuclear export [23]. Suppression of p38 kinase by a specific inhibitor has been shown to reduce IV replication [24]. On the basis of these facts, the pathogenic roles of NF-κB and p38 kinase in influenza diseases imply that these molecules might be suitable targets for influenza disease therapeutics.

AMP-activated protein kinase (AMPK) is a conservative serine/threonine kinase that plays a crucial role in maintaining energy homeostasis by promoting ATP generation [25]. Activation of AMPKα has the capacity to provide protection for cells in response to ATP depletion via biosynthetic pathway inhibition and catabolic metabolism pathway stimulation [25, 26]. In addition to its role in metabolism, there is evidence that AMPKα is involved in the induction of host immunity to bacteria and viruses [27]. AMPKα activation has been shown to participate in upregulation of stimulator of interferon genes (STING), which has an essential role in the recognition of viral and bacterial infection [28], and it has been reported that inhibition of AMPKα activity decreases the expression levels of interferon β (IFN-β), which directly impairs antiviral effects [28]. Moreover, AMPKα activation has been shown to ameliorate inflammation-related diseases through suppression of p38 MAPK and NF-κB signaling pathways [29]. On the basis of these observations, we hypothesized that stimulation with small-molecule activators of AMPKα activity may provide beneficial potential for influenza disease therapeutics.

Flavonoids, which are abundant in plants and provide many health benefits, have garnered wide public attention for their multiple biological activities, including anti-inflammation, antitumor, and antibacterial [30]. The diverse types of biological properties of flavonoids have been attributed to acting on a wide variety of therapeutic targets, such as NF-κB, MAPKs, and NRF2 [31]. In the context of IV infection, previous studies reported that flavonoids with Nrf2-activated property have been found to exert antiviral activity via upregulation of type I IFNs and IFN-stimulated genes (e.g., *IFIT1*, *PKR*, and *OAS2*) [32]. Moreover, flavonoids have the capacity to suppress IV-mediated NF-κB and MAPK signaling activation, thereby reducing IV replication and inflammation reactions [33, 34]. Notably, methoxyflavones were found to have superior biological activity and oral bioavailability to unmethylated flavonoids [35]. 5-Methoxyflavone (5-MF), a flavonoid compound with a methoxy group attached to the C5 atom, was reported to have chemopreventive potential for cancer development. However, the effects of 5-MF on influenza diseases have not been reported before. In this study, we hypothesized that 5-MF could protect against IAV-mediated lung injury and inflammation, and investigated the mechanism by which 5-MF protects against viral infection.

## Materials and methods

### Reagents and antibodies

**Fig. 1.**
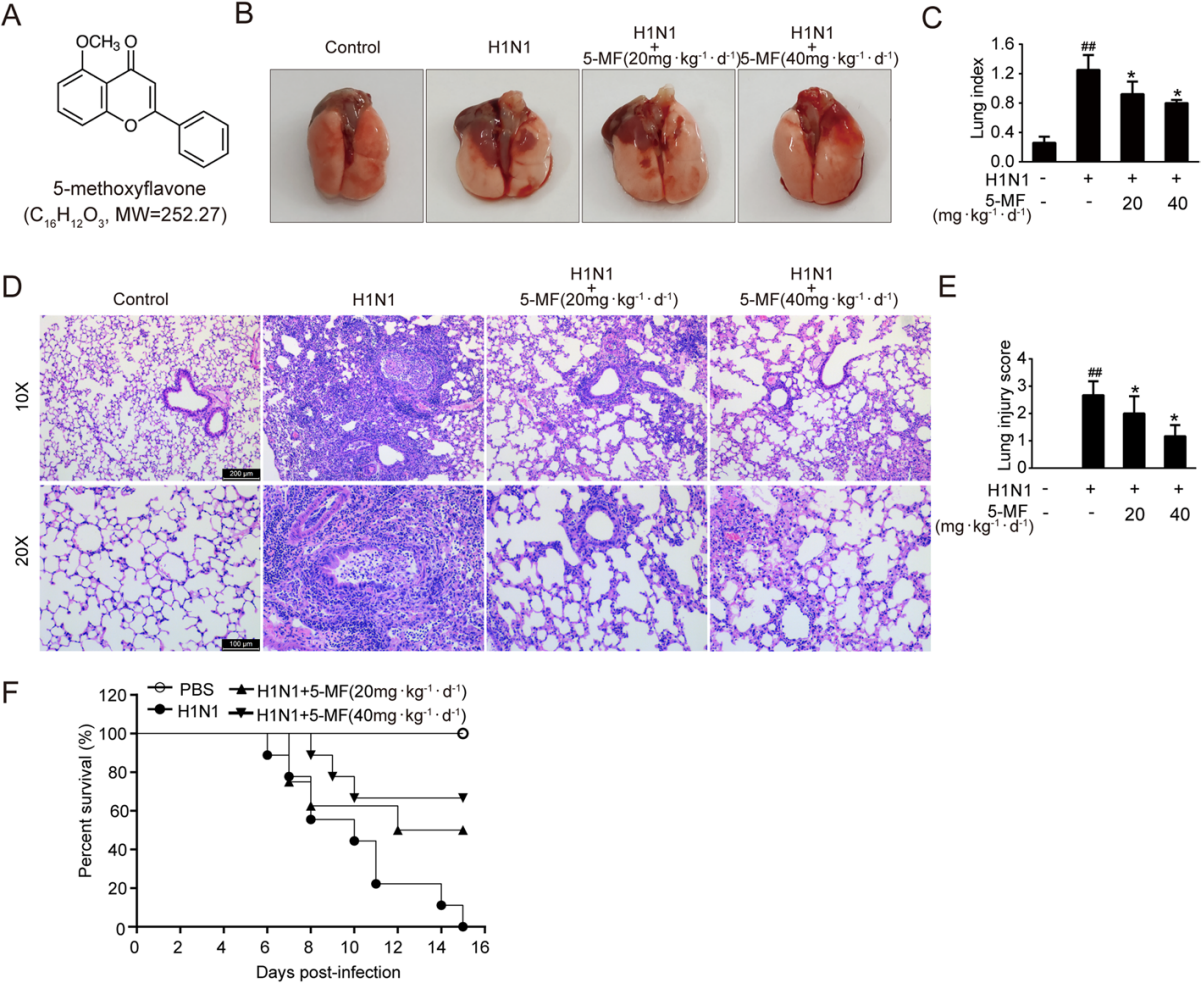
5-MF administration protected mice against IAV-induced lung injury. **A** Chemical structure of 5-MF. **B**–**F** Mice were intragastrically administered 5-MF (20, 40 mg·kg^−1^·d^−1^) 2 days prior to intranasal inoculation of 5LD_50_ of mouse-adapted H1N1 virus. **B**, **C** On day 5 post-infection (p.i.), the whole lung gross morphology of the mice was examined (**B**), and the lung index (*n* = 5) (lung/body weight ratio) was determined (**C**). **D** On day 7 p.i., histopathological changes of the lungs were examined by H&E staining. **E** Lung injury score was recorded according to the lung histopathology changes. **F** The survival rate of mice was observed and recorded for 15 consecutive days. ^##^*p* < 0.01 relative to the control group; ^*^p < 0.05 relative to the virus group

5-MF (purity > 98.0%) (Fig. 1A) was obtained from Tokyo Chemical Industry Co., Ltd. (Tokyo, Japan), and dissolved in DMSO to a stock concentration of 50 mM. Compound C was from MedChem Express (Shanghai, China). The multianalyte bead-based kit for determination of IL-6, CXCL8, TNF-α, CXCL10, CCL2, CCL3, CCL4, GM-CSF, and TRAIL was produced by Bio-Rad Laboratories Inc. (Hercules, CA, USA). The primary antibodies against phosphorylated-P65 (Ser^536^), P65, phosphorylated-P38 (Thr^180^/Tyr^182^), P38, phosphorylated-ERK1/2 (Thr^202^/Tyr^204^), ERK1/2, phosphorylated-P53 (Ser^15^), phosphorylated-JAK1 (Tyr^1034/1035^), JAK1, phosphorylated-STAT1 (Tyr^701^), STAT1, phosphorylated-STAT2 (Tyr^690^), STAT2, granzyme B, and COX-2 were purchased from Cell Signaling Technology (Beverly, MA, USA). Phosphorylated-IKBα (Ser^32^) and IKBα were provided by Beyotime Biotechnology Co., Ltd. (Shanghai, China). Phosphorylated-AMPK (Thr^172^), AMPK, CD8, EpCAM, cytokines for immunofluorescence (IL-6, TNF-α, CXCL8, and CCL2), and GAPDH were purchased from Affinity (Canal Fulton, OH, USA). RSAD2 and P53 were obtained from Proteintech (IL, USA).

### Cell culture

Madin-Darby Canine Kidney (MDCK) cells and human lung epithelial A549 cells were purchased from American Tissue Culture Collection (ATCC; Manassas, VA, USA), and cultivated in Dulbecco’s modified Eagle medium (DMEM) supplemented with 10% FBS and antibiotics (100 U/mL penicillin and 100 μg/mL streptomycin). These cells were grown as monolayers at 37 °C in a humidified 5% (v/v) CO_2_ atmosphere.

### Virus strains and viral infections

The IV strains, including A/PR/8/34 (H1N1) and A/HK/8/68 (H3N2), were purchased from American Type Culture Collection (ATCC; Manassas, VA, USA), and avian IV A/HK/Y280/97 (H9N2) was provided by Dr. Jing Li (State Key Laboratory of Respiratory Disease). All the virus strains were produced in 10-day-old chicken embryos, and titration of virus stocks was performed in MDCK cells by tenfold serial dilutions as described previously [36].

For viral infection, both the A549 and MDCK cells were washed twice with cold PBS and then inoculated with the indicated viruses diluted in serum-free culture medium. Two hours after virus absorption, fresh culture medium containing none or 2 μg/mL TPCK was added to A549 and MDCK cultures, respectively.

### Cytopathic effect inhibition assay

The antiviral effects of 5-MF were determined by cytopathic effect (CPE) inhibition assay in vitro as follows: MDCK cells (1 × 10^5^ cells per well, 100 μL) were seeded in 96-well plates and inoculated with 100TCID_50_ of IV at 37 °C for 2 h. Then, cells were washed twice with PBS to remove unabsorbed virus, and culture was continued in the presence of 5-MF (10–30 μM) in serum-free DMEM supplemented with 2 μg/mL TPCK. After 48 h, virus-induced CPE in MDCK cells was captured by light microscopy.

### Plaque reduction assay

Plaque reduction assay (PRA) was carried out as previously described [37]. In brief, confluent monolayers of MDCK cells in six-well tissue culture plates were infected with 100 plaque-forming units (PFU) per well of IV and incubated at 37 °C for 2 h. Afterward, the virus inoculums were discarded, and the cells were overlaid with a mixture of 200 µL per well of serum-free DMEM, 1% low-melting-point agarose, 2 μg/mL TPCK, and various concentrations of 5-MF. The culture plates were incubated at 37 °C for 2–3 days. Before the agarose layer was removed, the cell monolayers were fixed with 4% paraformaldehyde at room temperature for 2 h, and stained with 0.1% (w/v) crystal violet (Macklin, Shanghai, China).

### Acridine orange staining

To determine the effects of 5-MF on endosomal acidification, MDCK cells were stained with acridine orange as described previously [38]. Briefly, MDCK cells (2 × 10^5^ cells per well) were seeded in 20-mm-diameter glass-bottom dishes (Jet Bio-Filtration Co., Ltd, Guangzhou, China). After a 4 h pretreatment with 5-MF, cells were infected with influenza virus A/PR/8/34 (H1N1) (multiplicity of infection 0.1) for 1 h at 37 ℃. Afterward, cells were stained with acridine orange (4 μg/mL) for 10 min, and cells were analyzed using a Leica Stellaris confocal microscope (Leica, Wetzlar, Germany).

### Animal experiments

Four- to six-week-old specific-pathogen-free (SPF) C57BL/6 mice used in the present study were purchased from Guangdong Medical Laboratory Animal Center. The protocols for animal experiments were approved by the Animal Ethics Committees of the Affiliated First Hospital of Guangzhou Medical University. Mice were anesthetized with isoflurane and intranasally inoculated with 5LD_50_ of mouse-adapted H1N1 virus in 50 μL of PBS. Two days before viral intranasal inoculation, mice were intragastrically administered with 5-MF (20 or 40 mg·kg^−1^·d^−1^) or PBS (model group).

### Lung histopathology, lung pathological score, and immunofluorescence

At indicated timepoints, mice were euthanized, and the lungs were harvested and fixed in a 4% paraformaldehyde solution. After being embedded in paraffin, the lungs were cut into 4-μm sections. The histopathological alterations in the lung tissues were observed by hematoxylin and eosin (H&E) staining. The pathological scores of lung inflammation and injury were evaluated as previously described [39].

For immunofluorescence, lung tissue sections were deparaffinized and exposed to 0.01 mM citrate buffer (pH 6.0) for antigen retrieval. Then, the sections were blocked using 10% normal horse serum for 30 min at room temperature and incubated overnight at 4 °C with the indicated primary antibodies. Subsequently, the sections were incubated with fluorescein isothiocyanate (FITC)-conjugated goat anti-rabbit secondary antibodies (Multiscience, Hangzhou, China) for 1 h at room temperature. 4′,6-Diamidino-2-phenylindole (DAPI) staining solution (Beyotime, Shanghai, China) was applied to mark the nuclei. All sections were analyzed and captured by fluorescence microscopy (Leica, Wetzlar, Germany). The TSAPLus fluorescent triple staining kit obtained from Servicebio (Wuhan, China) was applied for multiple fluorescent immunolabeling of multiple antigens in the lung sections according to the manufacturer’s protocols. Terminal deoxynucleotidyl transferase dUTP nick end labeling (TUNEL) (Elabscience, Wuhan, China) analysis was performed according to the instructions of the manufacturer.

### Proinflammatory cytokine determination

The expression level of proinflammatory mediators in the culture supernatant was determined using the multianalyte bead-based kit (Bio-Rad Laboratories Inc., Hercules, CA, USA) according to the manufacturer’s protocols. The culture supernatant was collected and centrifuged at 10,000*g* for 10 min. After removing the cellular debris, the supernatant was aliquoted into stored at –80 °C until analysis.

### Western blotting

Total protein from A549 cells was extracted on ice by the addition of lysis buffer (Beyotime, China) containing protease inhibitors (Sigma, USA). Twenty micrograms of protein was heat denatured in sodium dodecyl sulfate loading buffer (Solarbio, Beijing, China), separated by 10% SDS–polyacrylamide gel electrophoresis, and transferred to PVDF membranes (0.2 μm, Bio-Rad, USA). Membranes were incubated overnight with the indicated primary antibodies, followed by incubation with HRP-conjugated secondary antibodies (Multi-science, Hangzhou, China). Protein bands were visualized by an ECL kit (Perkin Elmer Life Sciences). The original images for western blot are shown in Additional file 1.

### Flow cytometry

Apoptosis of IV-infected A549 cells was analyzed by an Annexin V-FITC detection kit (BD Biosciences, San Jose, CA, USA). Briefly, cells were detached by 0.5% EDTA-free trypsin and washed twice in 1 × binding buffer. Then, cells were resuspended in 100 μl 1× binding buffer, and stained with 5 μL Annexin V-FITC and 5 μL propidium iodide for 30 min under low-light environments. Cells were analyzed on a BD FACSCalibur flow cytometer.

### Statistical analysis

All data are presented as mean ± standard deviation (SD). All statistical analyses were performed using SPSS software version 18.0. Significant differences among groups were determined using one-way analysis of variance (ANOVA) followed by Newman–Student–Keuls tests, where *p* < 0.05 was considered statistically significant.

## Results

### 5-MF ameliorated IAV-mediated lung injury

To examine the prophylactic protective effects of 5-MF on IV-mediated lung injury in vivo, C57BL/6 mice were intragastrically administered with 5-MF 2 days prior to viral intranasal inoculation. After mice were challenged with 5LD_50_ of mouse-adapted influenza A/FM/1/47(H1N1) virus for 5 days, the results of gross anatomic pathology of the whole lung exhibited obvious pulmonary hemorrhage and edema, while 5-MF administration significantly improved these conditions (Fig. 1B). Simultaneously, the influenza H1N1 virus-elevated lung index, an indicator of lung injury, was also effectively reduced by 5-MF administration (Fig. 1C). Assessment of lung histopathological changes by H&E staining showed that influenza A (H1N1) virus-elicited lung tissue damage, including pulmonary parenchyma with diffuse inflammatory cell infiltration, bronchial edema with epithelial denudation, and the presence of inflammatory cells in the bronchioles, was significantly ameliorated by 5-MF administration (Fig. 1D). Consistently, 5-MF treatment decreased the lung pathological score (Fig. 1E). By day 15 p.i., untreated mice had succumbed to 5LD_50_ of influenza A (H1N1) virus infection, whereas mice receiving 5-MF at doses of 20 and 40 mg·kg^−1^·d^−1^ showed a prolonged survival rate of 50% and 66.7%, respectively (Fig. 1F). Therefore, these results suggested that 5-MF administration could provide protective effects against IAV-mediated lung injury.

### 5-MF suppressed IAV-triggered apoptosis

**Fig. 2:**
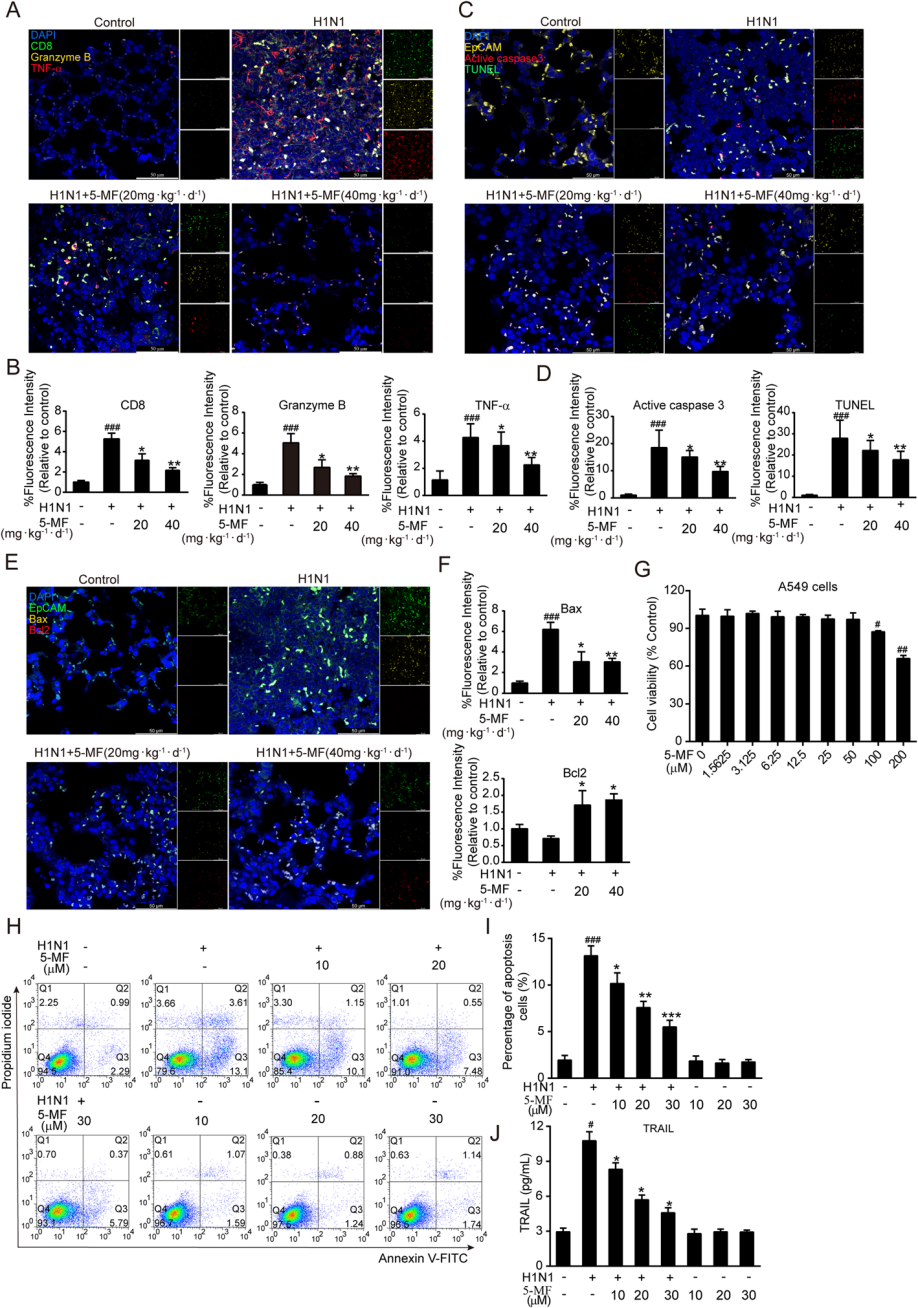
Effects of 5-MF on IAV-triggered apoptosis. **A** Immunofluorescence four-color staining showed colocalization of CD8^+^ (green), granzyme B (yellow), and TNF-α (red) in the lungs. Nuclei were stained with DAPI (blue). **B** The fluorescence intensities for granzyme B and TNF-α in CD8^+^ T cells were quantified. **C** Determination of apoptotic (green, TUNEL-positive; red, active caspase 3) lung epithelial cells (yellow, EpCAM) in the lungs by four-color immunofluorescence staining. **D** The fluorescence intensities for TUNEL and active caspase 3 were quantified. **E** Determination of the levels of Bax (yellow) and Bcl2 (red) in lung epithelial cells (green, EpCAM) in the lungs by four-color immunofluorescence staining. **F** The fluorescence intensities for Bax and Bcl2 were quantified. **G** The cytotoxicity of 5-MF (0–200 μM) on A549 cells was measured using the MTT assay. **H** H1N1 virus-infected A549 cells with 5-MF (0–30 μM) incubation for 24 h, and cell apoptosis in each group was detected by flow cytometry. **I** The percentage of apoptosis in H1N1 virus-infected A549 cells with or without 5-MF treatment. **J** Levels of TRAIL in H1N1 virus-infected A549 cells with or without 5-MF incubation were measured by Luminex assay. ^#^*p* < 0.05, ^##^*p* < 0.01, ^###^*p* < 0.001 relative to the control group; ^*^p < 0.05, ^**^p < 0.01, ^***^p < 0.001 relative to the virus group

Upon IAV infection, the progression of ALI can result from excessive cytotoxic CD8^+^ T-cell lung recruitment [1], and it is reported that the secretion of granzyme B and TNF-α by CD8^+^ T cells is responsible for the death of target cells [40]. Given that 5-MF protected against influenza A (H1N1) virus-elicited lung injury, we wondered whether 5-MF affected infiltration of CD8^+^ T cells and the expression pattern of CD8^+^ T cells in the lung tissue. Strikingly, the results of three-color immunofluorescence showed that the lung tissues of H1N1 virus-infected mice had higher numbers of CD8^+^ T cells with granzyme B and TNF-α colocalization when compared with those of H1N1 virus-infected mice with 5-MF administration (Fig. 2A, B). Because the epithelial cell apoptosis elicited by CD8^+^ T-cell lung infiltration has been linked to lung injury, we analyzed whether 5-MF could protect against epithelial cell apoptosis by staining of epithelial cell adhesion molecule (EpCAM, a non-cell-type-specific epithelial cell marker) with TUNEL and active caspase 3 colocalization. As expected, we observed that 5-MF administration could reverse the elevated frequency of EpCAM colocalized with TUNEL and active caspase 3 elicited by H1N1 virus infection (Fig. 2C, D). Meanwhile, 5-MF treatment exhibited decreased levels of pro-apoptotic factor Bax, and elevated the expression levels of anti-apoptosis factor Bcl2 in the lung tissues (Fig. 2E, F). Further, we performed in vitro experiments to detect the effects of 5-MF on H1N1 virus-mediated apoptosis of A549 cells by flow cytometry. To avoid the cytotoxicity of 5-MF eliciting apoptosis of A549 cells, we tested the toxicity of 5-MF on A549 cells by MTT assay. The results of MTT assay in Fig. 2G showed that treatment with 5-MF at concentrations up to 50 μM for 24 h did not affect the viability of A549 cells. Thus, the concentrations of 5-MF used in A549 cells ranged from 10 to 30 μM. As shown in Fig. 2H, I, the increased cell apoptosis of A549 cells caused by H1N1 virus was prevented by 5-MF treatment. Interestingly, 5-MF treatment suppressed the increased levels of apoptosis factor TRAIL in H1N1 virus-infected A549 cells (Fig. 2J). Therefore, these results suggested that 5-MF protected against H1N1 virus-induced ALI, which may result from suppression of granzyme B^+^ TNF-α^+^ CD8^+^ T-cell lung recruitment and epithelial cell apoptosis.

**Fig. 3:**
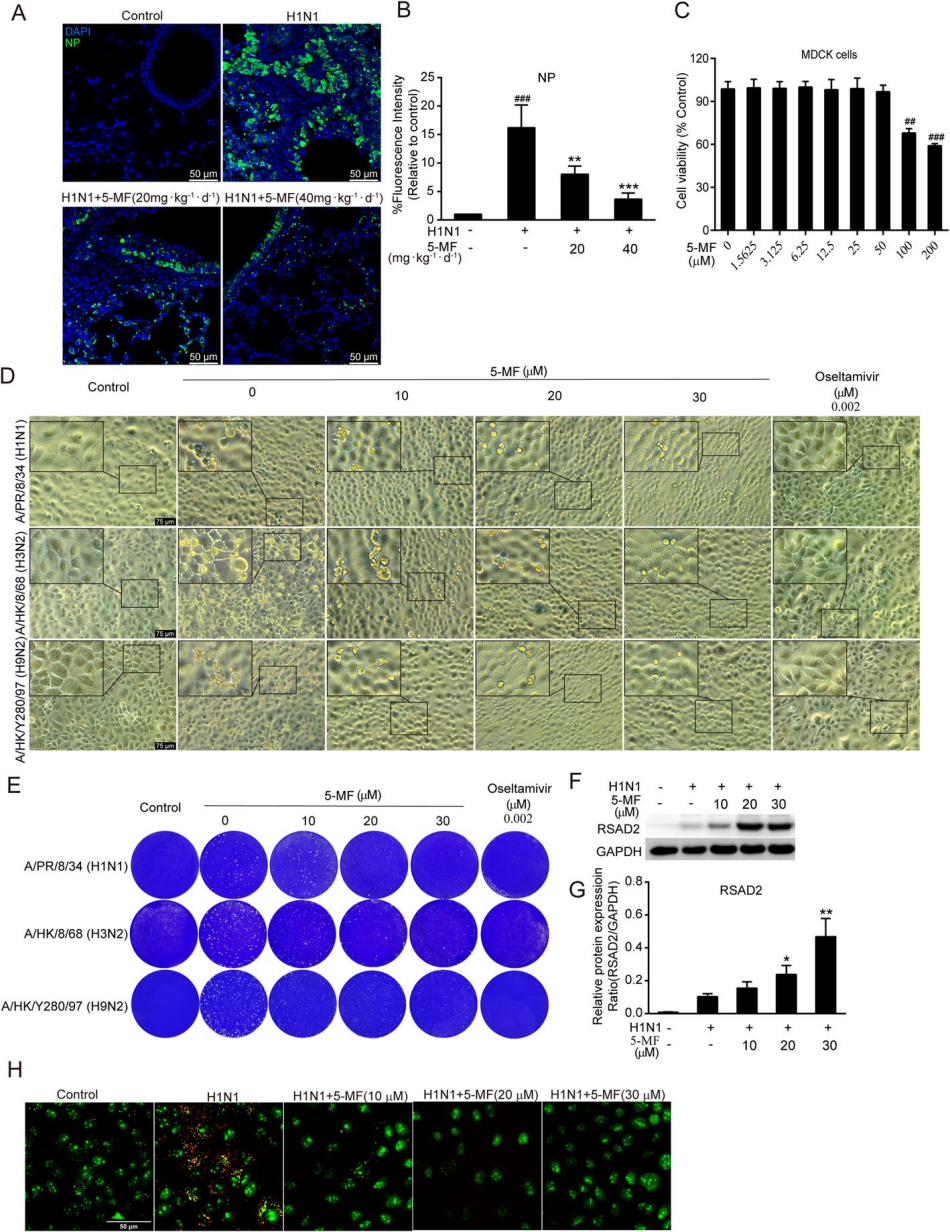
Anti-IAV activity of 5-MF. **A** Immunofluorescence analysis was performed to detect the expression of viral NP in the lung tissues. **B** The fluorescence intensity for viral NP protein was quantified. **C** The cytotoxicity of 5-MF (0–200 μM) on A549 cells was measured using the MTT assay. **D** CPE on MDCK cells induced by three IV strains [A/PR/8/34 (H1N1), A/HK/8/68 (H3N2), and A/HK/Y280/97 (H9N2)] were visualized using an inverted light microscope after a 48-h incubation. **E** 5-MF reduced IAV-induced the formation of viral plaques in MDCK cells. **F** Western blot analysis was performed to detect the expression of RSAD2 in the H1N1 virus-infected A549 cells. **G** The relative protein band intensity of RSAD2 was quantitated using ImageJ software. **H** MDCK cells were pretreated with 5-MF for 4 h, and then infected with H1N1 virus for 1 h at 37 °C. MDCK cells were stained with acridine orange (4 μg/mL) for 10 min and analyzed by confocal microscope. ^##^*p* < 0.01, ^###^*p* < 0.001 relative to the control group; ^*^p < 0.05, ^**^p < 0.01, ^***^p < 0.001 relative to the virus group

### 5-MF inhibited IAV replication in vitro and in vivo

High viral load is a critical contributor for the progression of ALI [41]. Next, we investigated the effects of 5-MF on the replication of IV in vivo and in vitro. Immunofluorescence staining of IV NP protein was found to be in the distribution of respiratory tract and lung parenchyma in the IV-infected group, whereas those were significantly decreased in 5-MF-administrated mice (Fig. 3A, B). Before examination of the antiviral activity of 5-MF in vitro, we determined the toxicity of 5-MF on MDCK cells by MTT assay. As shown in Fig. 3C, 5-MF did not affect the viability of MDCK cells at a concentration less than 50 μM. Therefore, we chose 30 μM of 5-MF as the maximum dose for its antiviral property determination. Cellular morphology of MDCK cells infected with three strains of IV [A/PR/8/34 (H1N1), A/HK/8/68 (H3N2), and A/HK/Y280/97 (H9N2)] observed under a microscope exhibited obvious cytopathic effects, including cell swelling, lysis and enlarged intercellular space (Fig. 3D), and these cytopathic effects were reduced upon treatment with 5-MF (Fig. 3D). Similarly, plaque reduction assay also demonstrated that 5-MF possessed antiviral activity against these IV strains (Fig. 3E). We wondered whether the antiviral effects of 5-MF were related to inducing the expression of antiviral effectors. Interestingly, we found that 5-MF treatment significantly increased the expression of RSAD2 (Fig. 3F, G). The acidic cytoplasmic compartment of virus-infected cells is required for the release of vRNPs into cytoplasm, and subsequent transfer into the nucleus for viral genome replication. Therefore, we wondered whether 5-MF affected the acidification of the cellular compartment during viral infection. As shown in Fig. 3H, the low pH in MDCK cells with acridine orange staining exhibited red fluorescence, which was weakened by 5-MF treatment. These results indicated that 5-MF elevated the pH of acidic cytoplasmic compartments, and thus prevented vRNP nuclear import. Taken together, our results demonstrated that 5-MF inhibited IV replication in vitro and in vivo, which might be attributed to its ability to raise the pH of acidic cytoplasmic compartments and thereby inhibit nuclear import of vRNPs.

### 5-MF decreased IAV-mediated inflammation

**Fig. 4.**
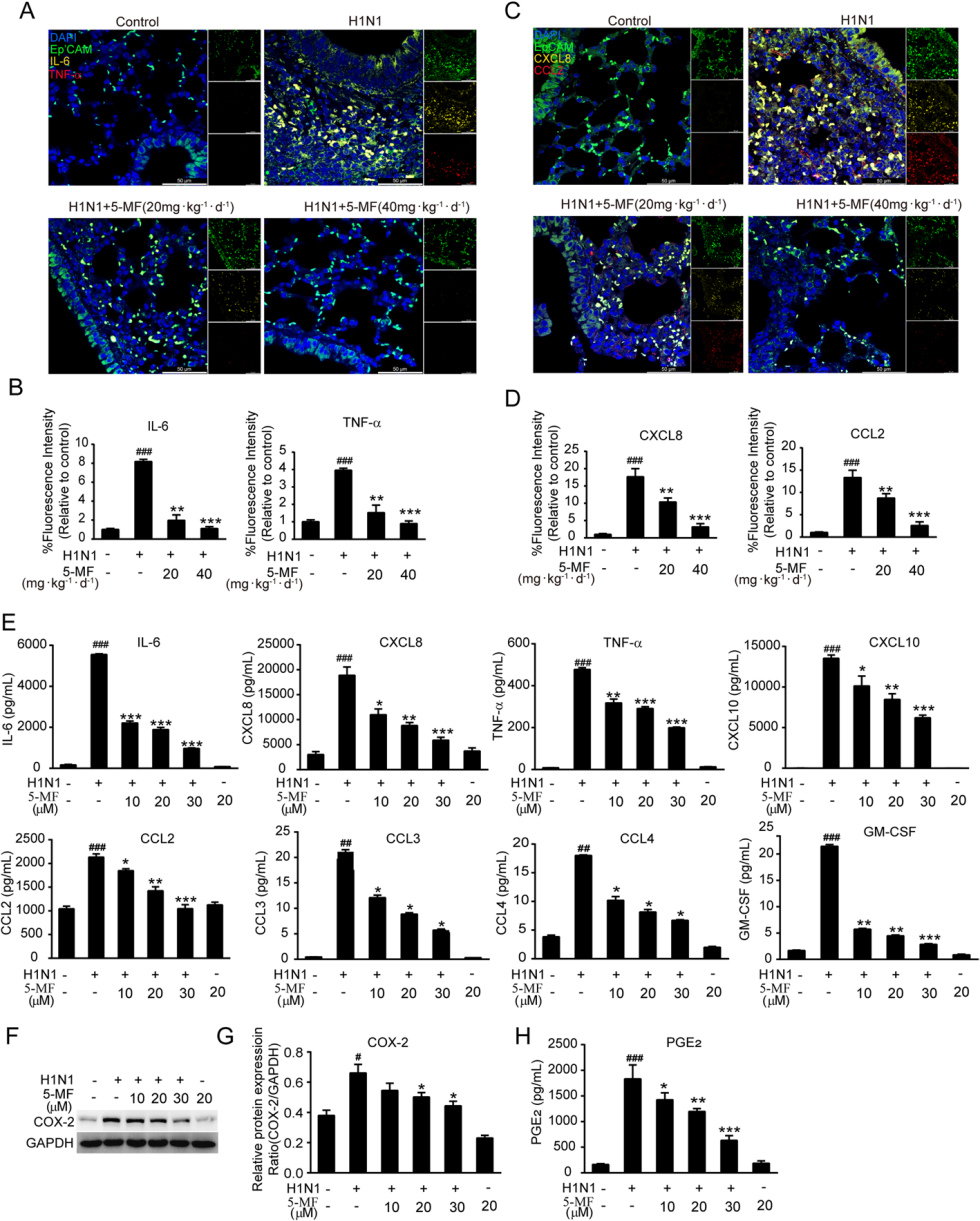
5-MF reduced H1N1 virus-elevated the expression levels of proinflammatory mediators. **A** Immunofluorescence four-color staining showed the expression of IL-6 (yellow) and TNF-α (red) in epithelial cells (detected by EpCAM; green). Nuclei were stained with DAPI (blue). **B** The fluorescence intensities for IL-6 and TNF-α were quantified. **C** Immunofluorescence four-color staining showed the expression of CXCL8 (yellow) and CCL2 (red) in epithelial cells (detected by EpCAM; green). Nuclei were stained with DAPI (blue). **D** The fluorescence intensities for CXCL8 and CCL2 were quantified. **E** Protein expression of a series of proinflammatory mediators (IL-6, CXCL8, TNF-α, CXCL10, CCL2, CCL3, CCL4, and GM-CSF) in the culture supernatants was analyzed by Luminex assay. **F** Protein expression of COX-2 was analyzed by western blotting. **G** The relative protein band intensity of COX-2 was quantitated using ImageJ software. **H** The levels of PGE_2_ in the culture supernatants were measured by ELISA assay. ^#^*p* < 0.05, ^##^*p* < 0.01, ^###^*p* < 0.001 relative to the control group; ^*^p < 0.05, ^**^p < 0.01, ^***^p < 0.001 relative to the virus group

Excessive inflammation in the lung epithelial cells is also associated with the severity and mortality of influenza diseases [42]. We therefore investigated the effects of 5-MF on virus-elicited inflammation in vitro and in vivo. The determination of the proinflammatory mediator expression with EpCAM colocalization by three-color immunofluorescence showed that 5-MF administration effectively reduced the expression of proinflammatory mediators (IL-6, TNF-α, CXCL8, and CCL2) in the lung epithelial cells of H1N1 virus-infected lung tissues (Fig. 4A–D). As shown in Fig. 3A–E, the results showed that 5-MF possessed antiviral activity. To rule out that the anti-inflammatory effects of 5-MF were not only due to its antiviral property, A549 cells were infected with H1N1 virus 2 h prior to 5-MF treatment. The effects of 5-MF on H1N1 virus-elicited inflammation in vitro were determined by Luminex assay. The results showed that 5-MF treatment dose-dependently reduced H1N1 virus-induced upregulated expression of proinflammatory mediators, including IL-6, CXCL8, TNF-α, CXCL10, CCL2, CCL3, CCL4, and GM-CSF (Fig. 4E). In addition, the increased levels of COX-2 and PGE_2_ induced by H1N1 viruses were decreased by 5-MF treatment (Fig. 4F–H). Therefore, these data demonstrated that 5-MF could reduce H1N1 virus-induced excessive inflammation in vitro and in vivo.

### 5-MF treatment suppressed IAV-mediated activation of NF-κB and P38 MAPK signaling pathways

**Fig. 5.**
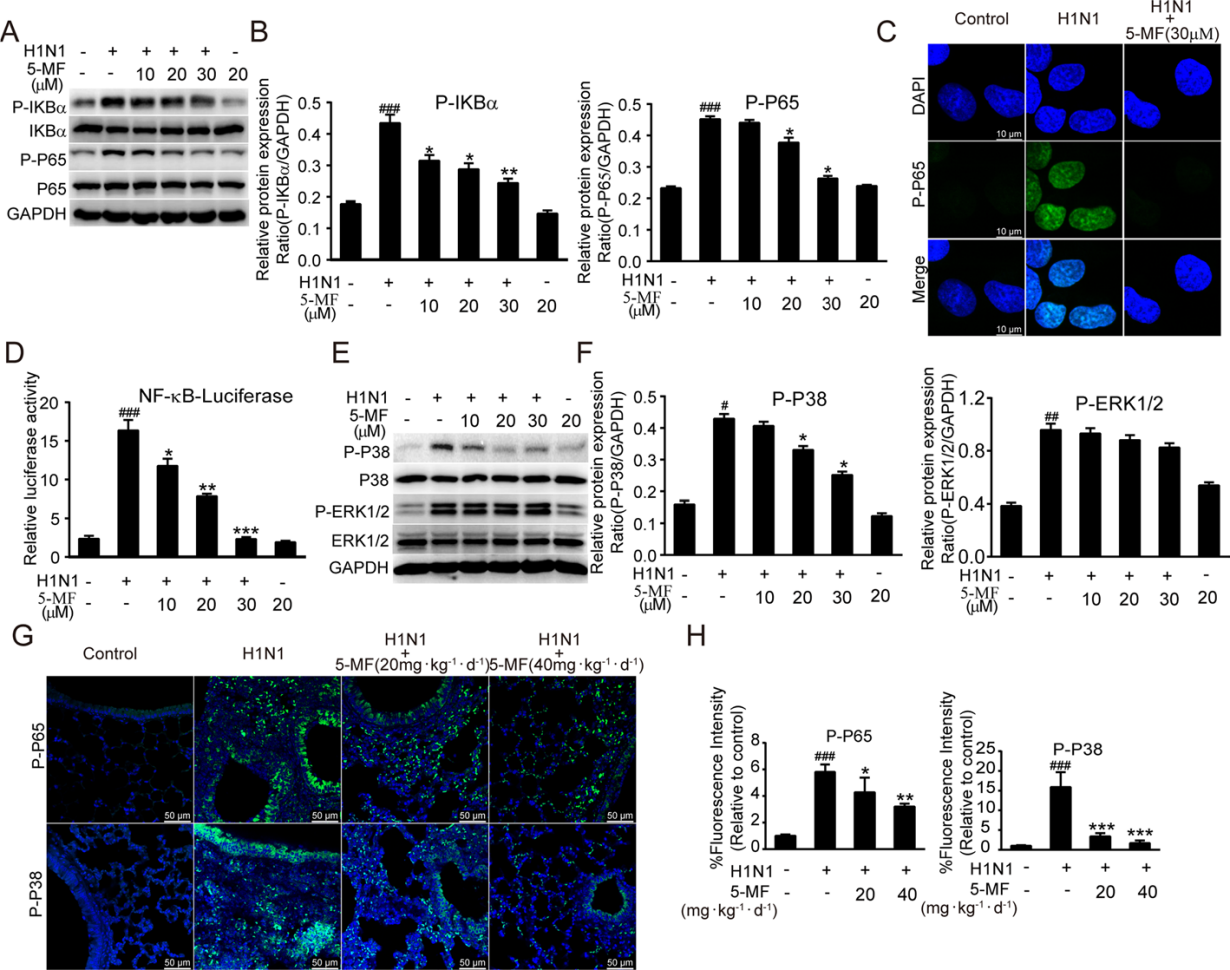
5-MF reduced the activation of NF-κB and P38 MAPK signaling upon H1N1 virus infection. **A** Western blotting was performed to measure the activation of NF-κB pathway in A549 cells. **B** The relative protein band intensities of P-IKBα and P-P65 were quantitated using ImageJ software. **C** The nuclear localization of P-P65 was detected by immunofluorescence. **D** A549 cells were transfected with an NF-κB-dependent luciferase reporter plasmid (pNFκB-TA-luc). At 12 h post-transfection, H1N1 virus-infected A549 cells were treated with or without 5-MF for 24 h, and then the luciferase activity was determined. **E** Western blotting was performed to detect the activation of MAPK pathways (P38 and ERK1/2) in H1N1 virus-infected A549 cells. **F** The relative protein band intensities for P-P38 and P-ERK1/2 were quantitated using ImageJ software. **G** On day 7 p.i., the lungs were harvested, and the activation of NF-κB (P-P65) and P-P38 MAPK in the lung tissues was detected by immunofluorescence. **H** The fluorescence intensities for P-P65 and P-P38 were quantified. ^#^*p* < 0.05, ^##^*p* < 0.01, ^###^*p* < 0.001 relative to the control group; ^*^p < 0.05, ^**^p < 0.01, ^***^p < 0.001 relative to the virus group

The activation of host cell signaling cascades is reported to be involved in IV replication and excessive inflammatory mediator production [13, 22]. We set out to investigate the effects of 5-MF on H1N1 virus-elicited host cell signaling transduction in host cells. Our results showed that H1N1 virus infection triggered increased phosphorylation levels of signaling molecules (P-IKBα and P-P65) of the NF-κB pathway in A549 cells, which were significantly inhibited by 5-MF treatment (Fig. 5A, B). Indeed, immunofluorescence staining showed that the nuclear translocation of P-P65 upon viral infection was abolished by 5-MF (Fig. 5C). Moreover, the effect of 5-MF on the transcriptional activity of NF-κB was measured by using an NF-κB luciferase reporter system, and the increased transcriptional activity of NF-κB following H1N1 virus infection was decreased by 5-MF (Fig. 5D). In addition, 5-MF treatment suppressed H1N1 virus-triggered activation of P-P38 MAPK but did not affect p-ERK1/2 activation (Fig. 5E, F). To verify whether 5-MF reduced H1N1 virus-mediated activation of NF-κB and P38 MAPK in vivo, immunofluorescence staining was performed to determine the levels of phosphorylated P65 and P38 in the tissues. In line with the in vitro data, 5-MF treatment reduced H1N1 virus-mediated phosphorylated P65 and P38 in vivo (Fig. 5G, H). These data suggested that reduction of NF-κB and P38 MAPK signaling activation by 5-MF probably played a critical role in alleviating viral replication and inflammation.

### Activation of AMPKα might be involved in the antiviral effects of 5-MF against IAV

**Fig. 6:**
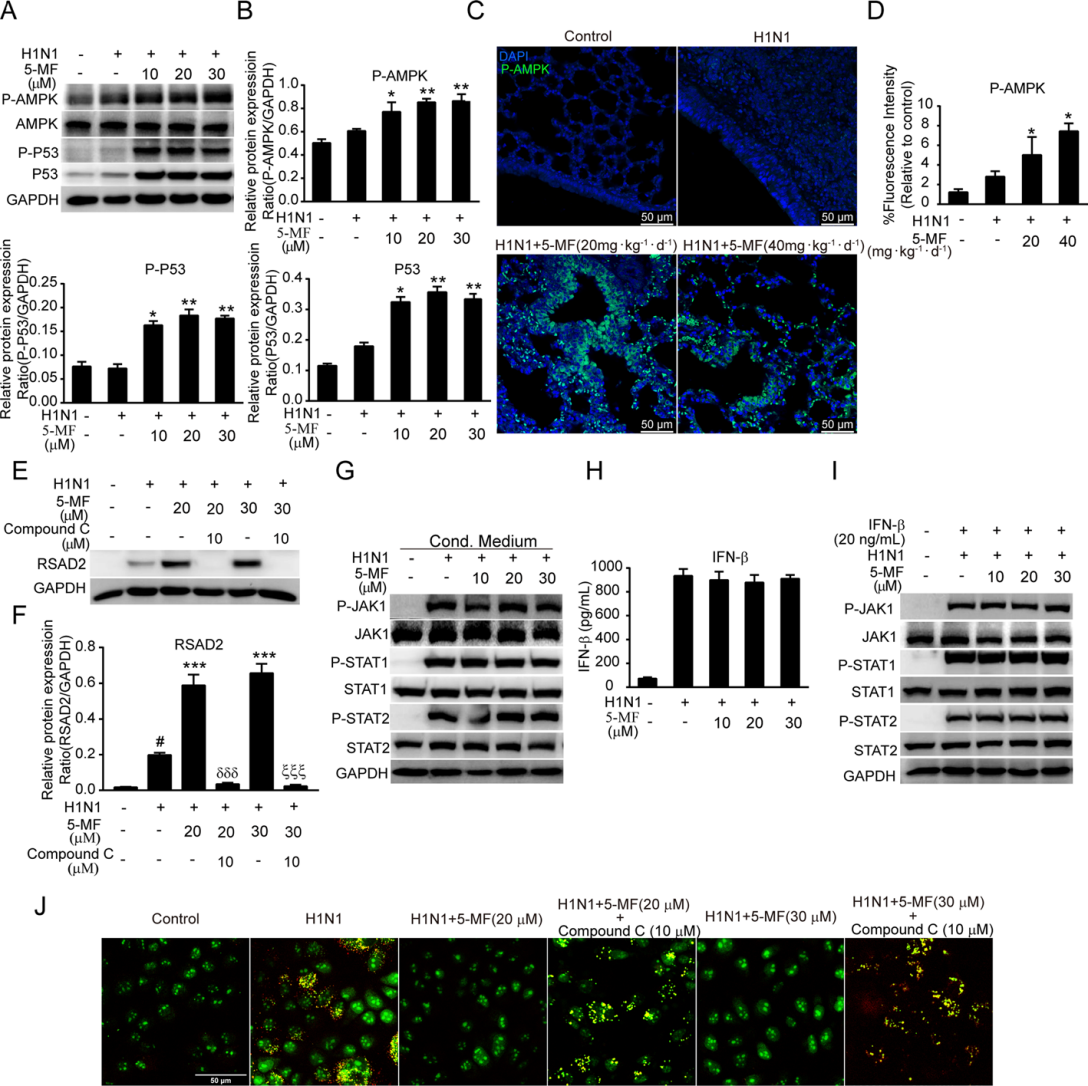
AMPKα was involved in the antiviral action of 5-MF. **A** Western blotting was performed to detect the expression of P-AMPKα, P-P53, and P53 in H1N1 virus-infected A549 cells. **B** The relative protein band intensities of P-AMPKα, P-P53, and P53 were quantitated using ImageJ software. **C** Immunofluorescence analysis was performed to detect the phosphorylation of AMPKα in the lung tissues. **D** The fluorescence intensity for P-AMPK was quantified. **E** H1N1 virus-infected A549 cells were pretreated with compound C (10 μM) for 30 min, followed by 5-MF treatment for 24 h. Western blotting was performed to analyze the expression of RSAD2. **F** The relative protein band intensity of RSAD2 was quantitated using ImageJ software. **G** The culture supernatants were collected from H1N1 virus-infected A549 cells with or without 5-MF 24 h treatment, and then transferred to uninfected A549 cells for 15 min of stimulation. Western blotting was performed to analyze the expression of P-JAK1, P-STAT1, and P-STAT2. **H** ELISA assay was performed to measure the levels of IFN-β in the culture supernatants. **I** A549 cells were infected with H1N1 viruses for 4 h, and then stimulated with recombinant human IFN-β (20 ng/mL) for 15 min. Western blotting was performed to analyze the expression of P-JAK1, P-STAT1, and P-STAT2. **J** After pretreatment with 5-MF alone or in combination with compound C (10 μM) for 4 h, MDCK cells were infected with H1N1 viruses for 1 h. Cells were stained with acridine orange (4 μg/mL) for 15 min and subsequently analyzed by confocal microscopy. ^#^*p* < 0.05 relative to the control group; ^*^p < 0.05, ^**^p < 0.01, ^***^p < 0.001 relative to the virus group; ^δδδ^*p* < 0.001 relative to the H1N1 virus + 5-MF (20 μM) group; ^ξξξ^*p* < 0.001 relative to the H1N1 virus + 5-MF (30 μM) group

AMPKα activation has been shown to be beneficial in reducing viral replication, which exerts biological effects through the P53 pathway [43]. Next, the effect of 5-MF on AMPKα activation was first analyzed in H1N1 virus-infected cells. Interestingly, immunoblotting results showed that the phosphorylation of AMPKα and P53 was significantly enhanced by 5-MF treatment (Fig. 6A, B). In addition, the augmented AMPKα phosphorylation was also observed in the lung tissues of H1N1 virus-infected mice with 5-MF administration (Fig. 6C, D). Given that 5-MF increased the expression of antiviral effector RSAD2 and AMPKα was found to be involved in RSAD2 expression [44], it was interesting to clarify whether the upregulation of RSAD2 by 5-MF was due to AMPKα activation. As expected, blockade of AMPKα by compound C abrogated 5-MF-mediated increased expression of RSAD2 (Fig. 6E, F). The classical upregulated expression of the antiviral effectors is dependent on type I IFN signaling transduction [45]. Therefore, we investigated whether 5-MF affected the expression of IFN-β and its downstream signaling. As shown in Fig. 6G, supernatant transfer experiments showed that 5-MF treatment did not enhance the activation of downstream signaling events of IFNs (Fig. 6G), including P-JAK1, P-STAT1, and P-STAT2, indicating that 5-MF did not alter the expression of IFNs. Indeed, ELISA assay demonstrated that there was no difference in IFN-β levels between 5-MF-treated and untreated cells in the culture supernatant (Fig. 6H). Moreover, upon IFN-β stimulation, the activation of P-JAK1, P-STAT1, and P-STAT2 was not augmented by 5-MF incubation (Fig. 6I). These results indicated that the increased expression of RSAD2 by 5-MF was independent of IFN signaling transduction. Interestingly, we found that blockade of AMPKα abolished the inhibitory effects of 5-MF on acidification of the endosomes (Fig. 6J). Altogether, these data indicated that AMPKα activation mediated by 5-MF treatment was responsible for the increased expression of RSAD2 as well as the reduction of endosome acidification, which resulted in the antiviral effects of 5-MF.

### Blockade of AMPKα abrogated the protective effects of 5-MF on H1N1 virus-mediated inflammation

**Fig. 7:**
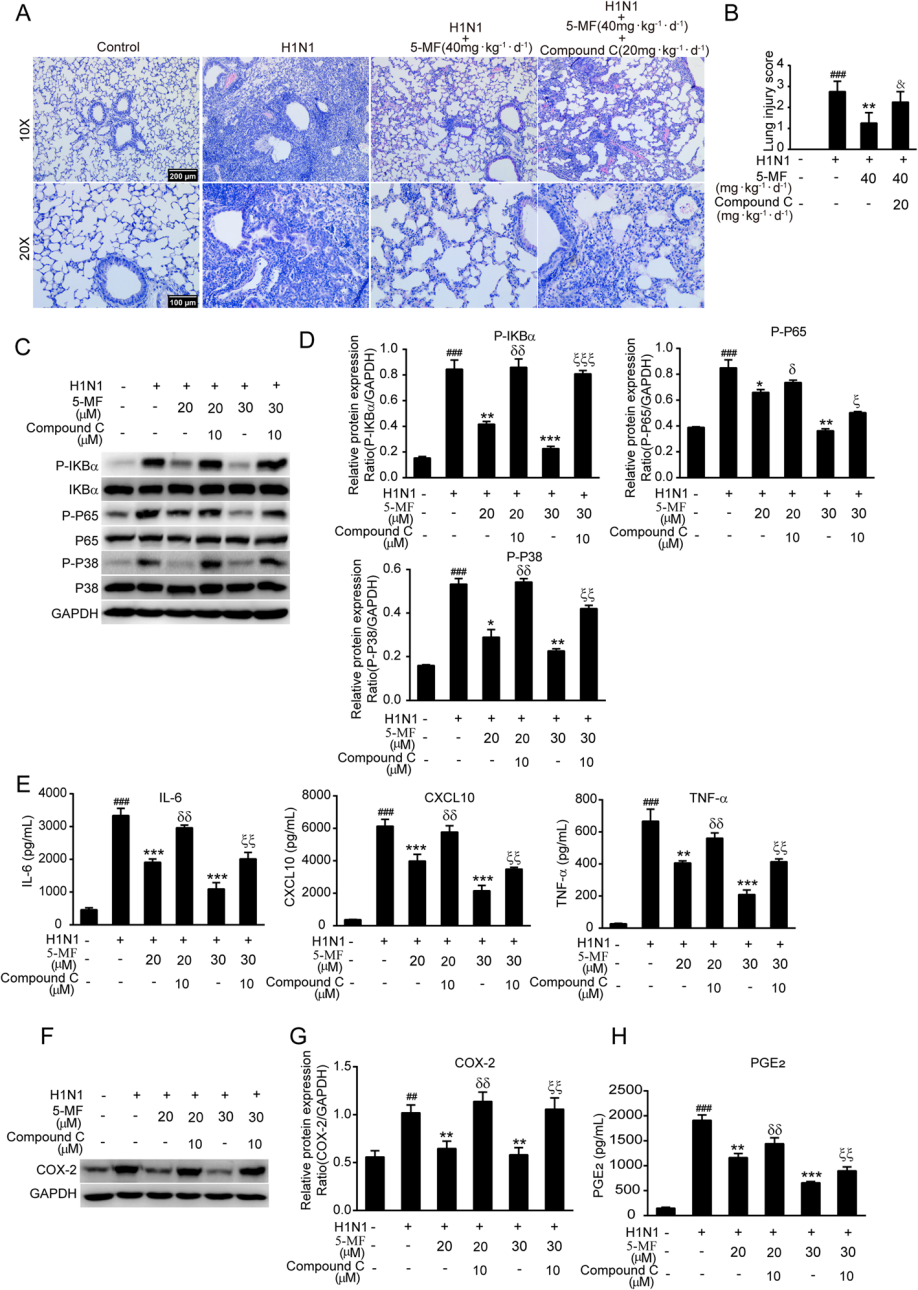
Activation of AMPKα by 5-MF has a role in the inhibition of NF-kB and P38 MAPK signaling pathways. **A** Two days prior to the 5LD_50_ of H1N1 virus infection, mice were intragastrically administered 5-MF alone or in combination with compound C (20 mg·kg^−1^·d^−1^) intraperitoneal injection, and the mice in the H1N1 + 5-MF + compound C group were intraperitoneally injected with compound C for 30 min before 5-MF administration. On day 7 p.i., histopathological changes of the lungs were examined by H&E staining. **B** Lung injury score was evaluated according to the lung histopathology changes. **C**–**H** After pretreatment with compound C (10 μM) for 1 h, H1N1 virus-infected A549 cells were incubated with 5-MF for 24 h. **C** Western blotting was performed to detect the phosphorylation of IKBα, P65, and P38. **D** The relative protein band intensities of P-IKBα, P-P65, and P-P38 were quantitated using ImageJ software. **E** Luminex assay for proinflammatory mediators (IL-6, CXCL10, and TNF-α) in culture supernatants. **F** Western blotting was performed to determine the expression of COX-2. **G** The relative protein band intensity of COX-2 was quantitated using ImageJ software. **H** ELISA assay for PGE_2_ in culture supernatants. ^##^*p* < 0.01, ^###^*p* < 0.001 relative to the control group; *p < 0.05, **p < 0.01, ***p < 0.001 relative to the virus group; ^&^*p* < 0.05 relative to the H1N1 virus + 5-MF (40 mg·kg^−1^·d^−1^); ^δ^*p* < 0.05, ^δδ^*p* < 0.01 relative to the H1N1 virus + 5-MF (20 μM) group; ^ξ^*p* < 0.05, ^ξξ^*p* < 0.01, ^ξξξ^*p* < 0.001 relative to the H1N1 virus + 5-MF (30 μM) group

Previous reports have been found that AMPKα activation could provide protective effects on IV-mediated lung injury [46]. Therefore, we hypothesized that the amelioration of H1N1 virus-mediated lung pathology by 5-MF was correlated with its activated effects on AMPKα. As shown in Fig. 7A, the improvement of 5-MF on H1N1 virus-elicited lung histopathological changes, including reduction of inflammatory cell lung parenchyma infiltration and alveolar destruction, was reversed in the lungs of mice with intraperitoneal injection with compound C. Accordingly, the lung pathological score also demonstrated that, in H1N1 virus-infected mice receiving compound C intraperitoneal injection, the ameliorative effects of 5-MF on H1N1 virus-mediated ALI were abolished (Fig. 7B). AMPKα activation has been shown to exert anti-inflammatory effects via inactivation of NF-κB and P38 MAPK signaling pathways [47]. In line with these findings, we speculated that activation of AMPKα by 5-MF may be involved in inhibition of NF-κB and P38 MAPK signaling pathways in H1N1 virus-infected cells. As shown in Fig. 7C, D, pretreatment with compound C reversed the inhibitory effects of 5-MF H1N1 virus-induced phosphorylation of IKBα, P65, and P38. Furthermore, compound C pretreatment abrogated the suppressive effects of 5-MF on H1N1 virus-induced production of the proinflammatory mediators, including IL-6, CXCL10, TNF-α, COX-2, and PGE_2_ (Fig. 7E–H). Therefore, these data suggested that 5-MF increased the phosphorylation of AMPKα, which attenuated the H1N1 virus-mediated activation of NF-κB and P38 MAPK signaling pathways, thus inhibiting H1N1 virus-elicited inflammation and lung injury.

## Discussion

Despite advances in management of infectious diseases, the morbidity and mortality of patients with influenza-related lung injury remains high, primarily owing to viral resistance of virus-targeting drugs and overactive host immune responses. Therefore, continued efforts to identify agents that efficiently treat influenza diseases are extremely essential. In our study, we investigated the effects of 5-MF on IV-mediated lung injury and inflammation in vitro and in vivo, and also sought to clarify a possible corresponding mechanism. Our results showed that 5-MF exerted protective effects against IV-mediated lethal lung injury, probably due to its antiviral and anti-inflammatory properties. Mechanistic studies showed that 5-MF treatment upregulated expression of antiviral effector RSAD2, inhibited endosomal acidification, and suppressed NF-κB and P38 MAPK signaling pathways via AMPKα activation, which in turn impaired IV replication and excessive proinflammatory mediator production, respectively.

Several studies have reported that 5-MF possesses various pharmacological properties, including antitumor, neuroprotective, and gastroprotective, which are attributed to its ability to act on multiple molecular targets, such as p53, p21, and β-amyloid [48–50]. However, there have been few studies demonstrating the effects of 5-MF on IV replication during IV-mediated lung injury. In our study, we have identified that AMPKα activation was involved in the inhibitory effects of 5-MF on IV replication as well as the protective effects against IV-mediated lung injury. Although AMPKα plays a critical role in the regulation of energy homeostasis, it has been found to promote antiviral immunity in response to viral infection [28, 51, 52], and AMPKα activation restricts viral replication via upregulation of the antiviral effector RSAD2 [44]. Consistent with these findings, the expression levels of RSAD2 were increased by 5-MF treatment, and CPE inhibition assay and plague reduction assay demonstrated that 5-MF was found to possess antiviral activity against several IV strains, including A/PR/8/34 (H1N1), A/HK/8/68 (H3N2), and A/HK/Y280/97 (H9N2). Moreover, the anti-IV property of 5-MF was further confirmed by the detection of viral antigens in the lung, which were significantly reduced by 5-MF treatment. The classical signaling pathway for RSAD2 induction in response to viral infection is dependent on type I IFN signaling transduction. Interestingly, we found that 5-MF treatment did not increase the expression levels of IFN-β or activate its downstream signaling molecules (P-STAT1 and P-STAT2), while blockade of AMPKα by compound C effectively repressed the expression of 5-MF-induced RSAD2, which was consistent with previous findings that AMPKα exerted antiviral activity independent of type I IFN signaling [51]. These data indicate that the protective effects of 5-MF against IV-mediated lung injury may be associated with reduction of viral replication through a mechanism involving activation of AMPKα/RSAD2.

Excessive inflammation also plays a critical role in determining the outcome of IV-associated lung injury [53]. Therapeutic strategies that modulate the host immune response, rather than targeting solely on the virus, are gaining appeal. Our results showed that 5-MF reduced IV-induced recruitment of CD8^+^ T cells with increased granzyme B and TNF-α expression in the lungs. Although IV-specific CD8^+^ T cells are believed to be responsible for eliminating viral infection, the activity of these cells should be tightly regulated as they are likely to cause damage to uninfected cells [40]. Several studies revealed that CD8^+^ T-cell-derived granzyme B and TNF-α were implicated in IV-mediated lung injury [40, 54], and inhibition of TNF-α signaling in CD8^+^ T cells markedly relieved lung injury after IV infection [54]. Therefore, we hypothesized that the reduction of CD8^+^ T-cell recruitment along with granzyme B and TNF-α expression by 5-MF may be sufficient to alleviate IV-induced lung injury to some extent. Furthermore, we observed that 5-MF suppressed the IV-induced expression of a series of proinflammatory cytokines (IL-6, CXCL8, TNF-α, CXCL10, CCL2, CCL3, CCL4, and GM-CSF) in vitro and in vivo. The dysregulation of cytokines was found to be correlated with fatal outcomes in critically ill patients with IV infection [41]. Although cytokines play a role in controlling viral spread, too many of them might exacerbate the progression of morbidity and lung injury [41]. Immunomodulatory drugs have been found to ameliorate IV-induced immunopathology by suppressing excessive cytokine production [55, 56]. Blockade of TNF-α can provide protection against severe lung injury induced by lethal IV infection [57]. Increased levels of IL-6 are directly correlated to influenza symptoms [58]. Excessive TNF-α disturbs the tight junction barrier of alveolar epithelia as well as endothelia, thus promoting the development of lung injury [59, 60]. NF-κB and P38 MAPK are critical regulators involved in the regulation of proinflammatory cytokines, which can be activated by viral infection or several cytokines (e.g., TNF-α and IL-1β) [10]. It has been reported that both NF-κB and P38 kinase signaling cascades play pivotal roles in robust cytokine production during low or high pathogenic IV infection [13, 21]. Blocking NF-κB or P38 kinase signaling has been found to alleviate excessive proinflammatory cytokine secretion and improve lethal IV-triggered lung injury [37]. Our results show that 5-MF treatment effectively decreased H1N1 virus-mediated NF-κB and P38 kinase signaling in vitro and in vivo. Therefore, we suggest that the protective effects of 5-MF against IV-mediated lung injury are associated with its anti-inflammatory properties, resulting from inhibition of IV-mediated activation of NF-κB and p38 MAPK signaling cascades.

The role of AMPKα in innate immune modulation has been less explored than its role in metabolism, and conflicting findings remain. There is evidence that activation of AMPKα by compounds (e.g., metformin and resveratrol) is linked with their anti-inflammatory properties [61, 62]. Recent studies have shown that AMPKα agonists can provide beneficial effects in allergy or lethal IV-mediated lung injury by reducing inflammation [46, 61–63]. Thus, AMPKα may be a promising target in the development of novel drugs for ameliorating influenza-associated inflammation and lung injury. To further elucidate the functional mechanism of 5-MF, we hypothesized that the modulation of IV-mediated immune response by 5-MF was correlated with its AMPK-activated effects, and blockade of AMPKα by compound C dramatically abrogated the alleviated effects of 5-MF on IV-mediated lung injury and pathological changes. In addition, it has been reported that AMPKα activation has the capacity to lower inflammation reactions via suppression of NF-κB and MAPK (P38 and JNK) signaling pathways [64, 65]. With further evidence clearly demonstrating that AMPKα could affect proinflammatory response, both NF-κB and MAPK (P38 and ERK1/2) were engaged in boosting proinflammatory cytokine production in AMPK-deficient cells [66]. Consistent with these findings, our results revealed that inhibition of AMPKα abolished the inhibitory effects of 5-MF on IV-induced activation of NF-κB and P38 MAPK signaling pathways as well as proinflammatory mediators. It has been reported that AMPKα activation exerts immunoregulatory functions mainly through several pathways, including p53/FoxO, SIRT1, and PGCα1 [43]. We found that the upregulation of AMPKα by 5-MF treatment was accompanied by increased expression of P53, indicating that P53 as a downstream effector of AMPKα signaling exerted the pharmacological protective effects of 5-MF during IV infection. In fact, P53 deficiency has been reported to enhance NF-κB activity, which has been associated with aberrant inflammatory responses and oxidative stress [67]. Previous reports have demonstrated that P53 has the capacity to modulate excessive inflammation via inhibition of NF-κB and P38 MAPK. On the basis of these findings, we conclude that 5-MF activates AMPKα/P53 signaling to suppress IV-mediated activation of NF-κB and P38 MAPK signaling, which results in a reduction of excessive inflammation.

## Supplementary Information

**Additional file 1.** Original images for western blot.

## Data Availability

The datasets used and/or analyzed during the current study are available from the corresponding author on reasonable request.

## Abbreviations

ALI: Acute lung injury
EpCAM: Epithelial cell adhesion molecule
H&E: Hematoxylin and eosin
IAV: Influenza A virus
IV: Influenza virus
MAPKs: Mitogen-activated protein kinases
NF-κB: Nuclear factor κB
RSAD2: Radical *S*-adenosyl methionine domain containing 2
TUNEL: Terminal deoxynucleotidyl transferase dUTP nick end labeling
