# Supplementary figures and images for: 5-Methoxyflavone-induced AMPKα activation inhibits NF-κB and P38 MAPK signaling to attenuate influenza A virus-mediated inflammation and lung injury in vitro and in vivo
Source: Cell Mol Biol Lett. 2022 Sep 30;27:82. doi: 10.1186/s11658-022-00381-1 (PMC9524045; doi:10.1186/s11658-022-00381-1)

Figure 3E

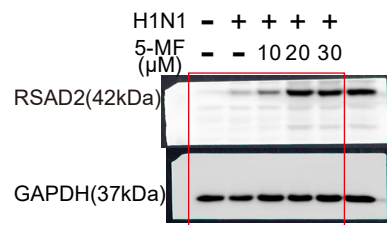

Figure 4F

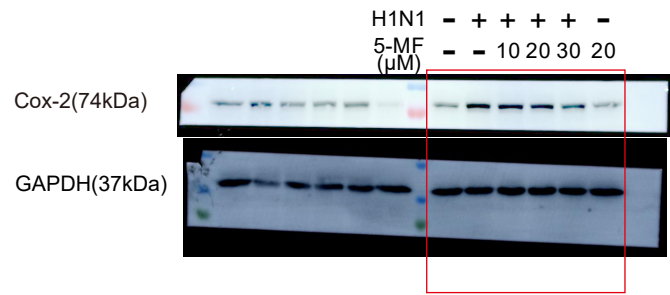

Figure 5A

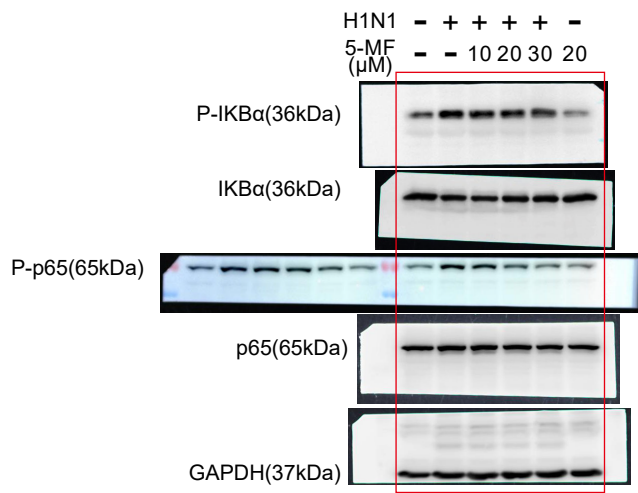

Figure 5E

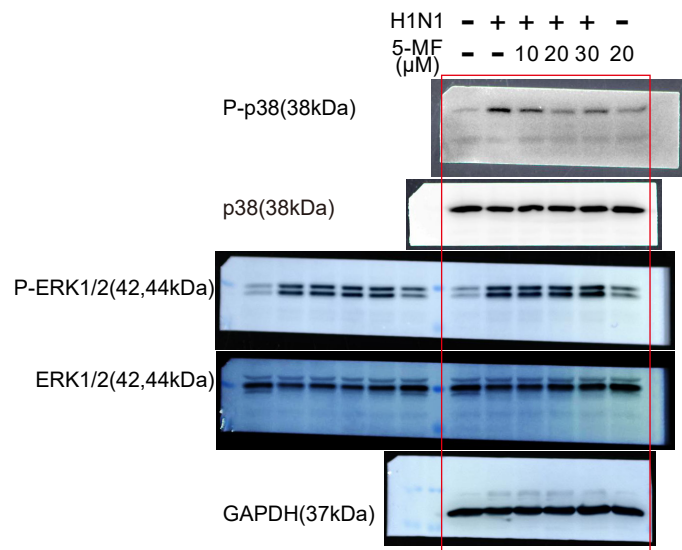

Figure 6A

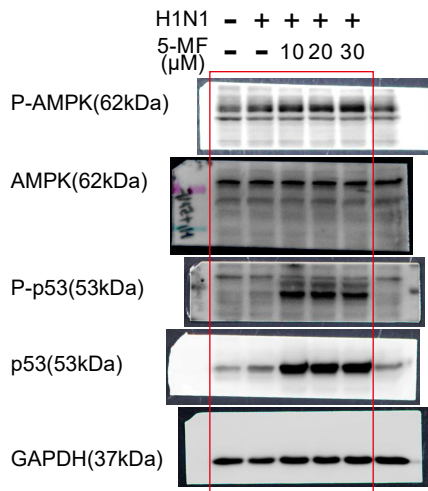

Figure 6E

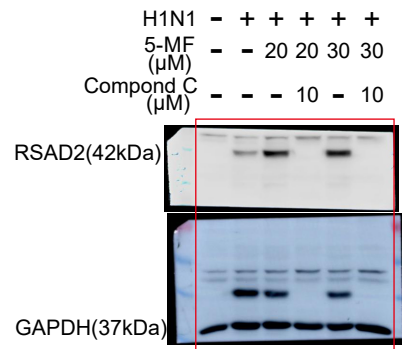

Figure 6G

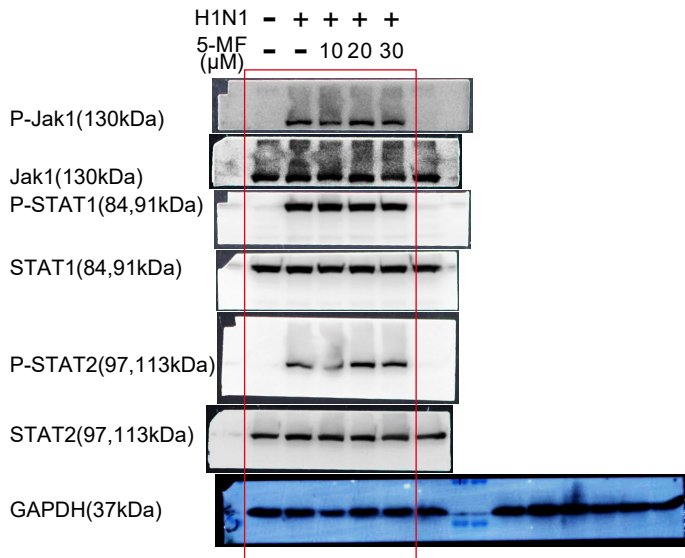

Figure 6I

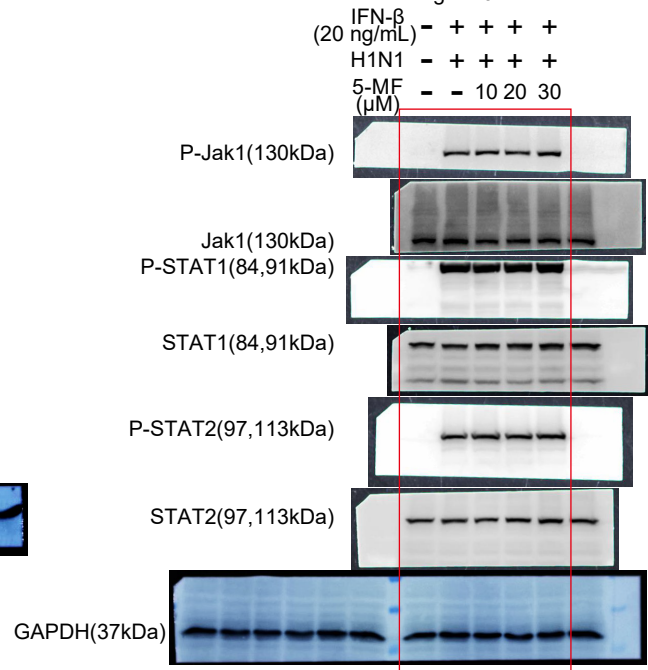

Figure 7C

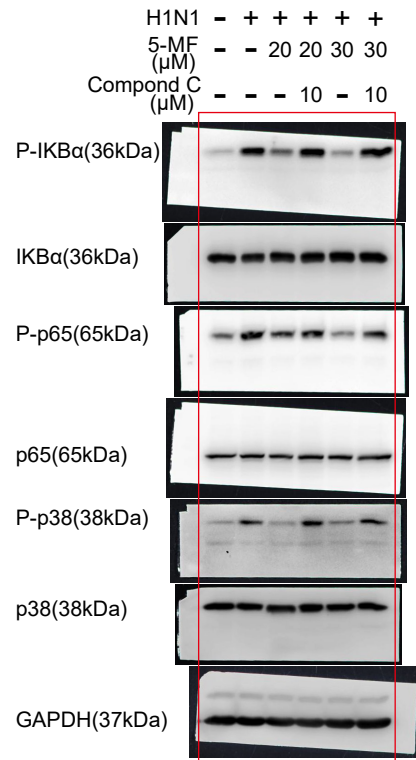

Figure 7F

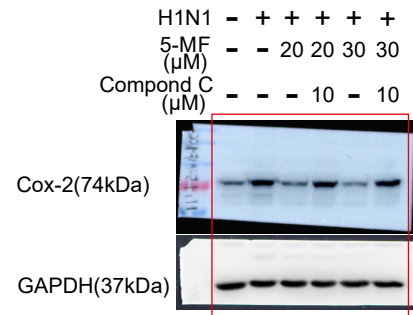

Supplement: Supplementary file 1 — Additional file 1. Original images for western blot. [file 11658_2022_381_MOESM1_ESM.pdf]
